# Biomass-Derived Cellulose Acetate Membranes Modified with TiO_2_/Graphene Oxide for Oil-In-Water Emulsion Treatment

**DOI:** 10.1021/acsomega.4c05980

**Published:** 2024-09-16

**Authors:** Djanyna V. C. Schmidt, Tainara L. G. Costa, Daniel F. Cipriano, Carla S. Meireles, Cleocir J. Dalmaschio, Jair C. C. Freitas

**Affiliations:** † Laboratory of Carbon and Ceramic Materials, Department of Physics, Federal University of Espírito Santo, 29075-910 Vitória, Espírito Santo, Brazil; ‡ Laboratory of Advanced Materials, Department of Natural Sciences, Federal University of Espírito Santo, 29932-540 São Mateus, Espírito Santo, Brazil; § Laboratory of Polymers, Department of Chemistry, Federal University of Espirito Santo, 29075-910 Vitória, Espírito Santo, Brazil

## Abstract

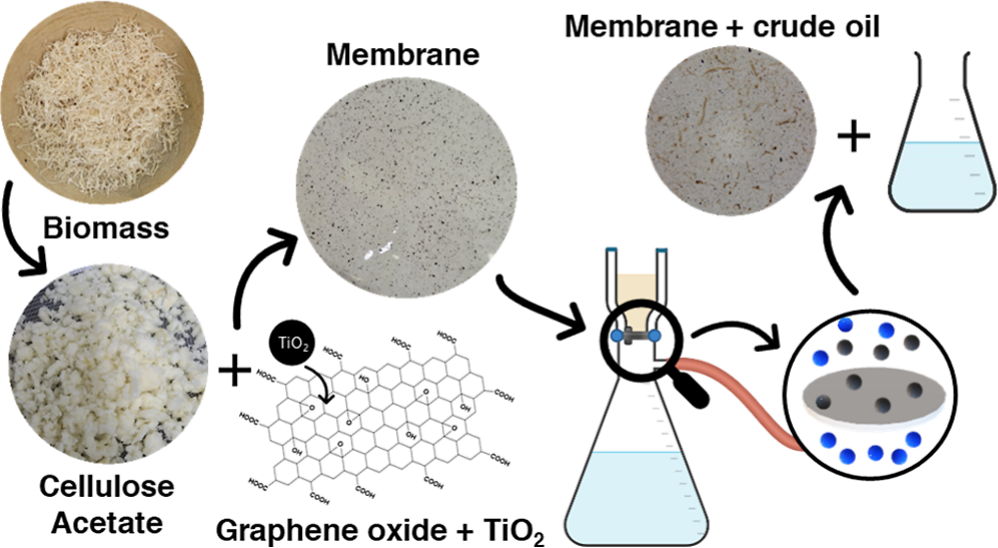

Considering the environmental impact and health risks caused by oily wastewater in the petrochemical industry, it is crucial to develop more efficient separation techniques than traditional methods, such as membrane separation, for treating stable emulsions enriched with natural surfactants. This study investigated the preparation of dense cellulose acetate membranes from a low-cost biomass precursor (*Luffa cylindrica*) and their modification with graphene oxide (GO) and TiO_2_ nanoparticles, aiming to obtain a polymeric nanocomposite with good flux characteristics and selectivity for the treatment of oil/water emulsions. The materials obtained were characterized using techniques such as X-ray diffraction, nuclear magnetic resonance spectroscopy, infrared absorption spectroscopy, along with optical and scanning electron microscopy, among others. The membranes were prepared by the casting technique and modified with the above-mentioned nanostructured materials. Flux analyses with petroleum emulsion revealed that membranes modified with GO and TiO_2_ nanoparticles showed significant improvements in antifouling resistance compared to unmodified membranes. These enhanced properties highlight the potential of modified cellulose acetate membranes for application in industrial wastewater treatment.

## 1 Introduction

With the development of petrochemical industries, oily wastewater has become a significant environmental and health concern.^1,2^ Typical methods for separating oil and water, such as centrifugation and flotation, often exhibit low efficiency when dealing with emulsified oily wastewater.^3^ Emulsions in the petroleum industry are particularly stable due to surfactants such as resins and asphaltenes.^4^ In addition, conventional methods for separating emulsions, such as the use of demulsifiers, can cause secondary pollution and have low efficiency.^5,6^ Therefore, there is a growing need for new separation techniques, such as the membrane separation processes.

Membrane separation can be highly efficient in emulsion separation without the need for chemical additives.^5,6^ The use of polymeric materials for membrane fabrication is attractive because they are more resistant and offer high performance in separation processes.^7^ Polymers such as polyvinylidene fluoride,^8^ polysulfone (PSF),^9^ poly(ether imide) (PEI),^10^ poly(ether sulfone) (polyvinyl chloride (PES)),^11^ polyvinyl chloride (PVC),^12^ polyacrylonitrile (PAN),^13^ and cellulose acetate^14^ are commonly used in membranes for separation processes.

Lignocellulosic precursors derived from agro-industries constitute a promising renewable resource, which is basically composed of three biopolymers: lignin, hemicellulose and cellulose.^15^ Cellulose, the most abundant biopolymer in nature, is predominantly found in plant cell walls.^16^ However, due to the presence of hydroxyl groups in the cellulosic chain formed by β-1,4-glycosidic bonds, there are intra- and intermolecular hydrogen bond interactions. As a consequence, cellulose is poorly soluble in most solvents, including water.^17,18^ On the other hand, these hydroxyl groups also facilitate the surface modification of cellulose and the modification of its physicochemical properties.^19^ Cellulose acetate is a widely used cellulose derivative, mostly because it is a biodegradable and nontoxic^20,21^ polymer that is soluble in different solvents,^22^ making it attractive for membrane manufacture. However, cellulose acetate membranes face challenges such as compaction under high pressures^23^ and fouling,^24^ which is the process of depositing or adsorbing particles on the membrane surface. This phenomenon shortens the membrane lifetime, reduces flux and modifies selectivity during the filtration process,^25^ representing one of the greatest challenges in the field of semipermeable membranes.

The incorporation of nanostructured materials into polymer matrices can significantly improve properties such as permeability, selectivity, mechanical strength and fouling resistance, thereby improving separation efficiency.^26,27^ The use of graphene oxide (GO) in polymeric matrices can improve their mechanical properties, increase their hydrophilicity, and reduce fouling by organic materials.^28^ Zhao et al.^29^ incorporated GO into PVC membranes and observed an increase in the hydrophilicity, water flux, and antifouling properties of the membrane. Nevertheless, due to the number of oxygenated groups on the surface of GO, there may be incomplete and irregular dispersion in the polymer matrix.^28,30^ However, these oxygenated groups also allow the chemical modification of the GO surface, making its properties adjustable.^30^ Safarpour et al.^31,32^ modified the reduced graphene oxide (rGO) surface with titanium oxide (TiO_2_) nanoparticles and incorporated them into PES^31^ and PVDF^32^ polymeric matrices, improving the hydrophilicity, water flux and antifouling properties of the membranes made from both polymeric materials. In addition, surface functionalization of nanoparticles with organic layers can control nanoparticle growth^33^ and improve nanoparticle stability within the polymer matrix.^33^ Parvizian et al.^34^ functionalized the surface of TiO_2_ with oleic acid and applied it to PES membranes, resulting in increased membrane hydrophilicity and water flux.

This study investigates the preparation of cellulose triacetate from an alternative low-cost biomass precursor that was purified to obtain cellulose before acetylation. Subsequently, pure and modified dense membranes were prepared using the cellulose triacetate. GO and GO functionalized with TiO_2_ nanoparticles were used as modifiers in the membranes. Following preparation, the membranes were evaluated for their flux performance and selectivity in separating oil/water emulsions. The results of the flux tests revealed that the GO/TiO_2_-modified membranes exhibited significant improvements in antifouling resistance compared to the unmodified membrane, highlighting the potential of modified cellulose acetate membranes for application in industrial wastewater treatment.

## 2 Materials and Methods

### 2.1 Materials

All reagents were used as received and were purchased from local suppliers: HNO_3_ (ACS, 70%); ethanol (Sigma-Aldrich, P.A.); NaOH (NEON, P.A.); H_2_SO_4_ (ACS, 98%); glacial acetic acid (ACS); acetic anhydride (ACS, P.A.); NaOH (NEON, P.A.); HCl (NEON, 37%), dichloromethane (Sigma-Aldrich, 99.8%); NANO_3_ (NEON, P.A.); KMnO4 (NEON, P.A.); H_2_O_2_ (ACS, 30%); CHCl_3_ (Sigma-Aldrich, 99%); oleic acid (NEON, P.A.); titanium butoxide (IV) (Sigma-Aldrich, 97%); Triton-X100 (Sigma-Aldrich); hexane (Sigma-Aldrich, 95%); anhydrous sodium sulfate (Dynamics, P.A.); and dichloromethane-d_2_ (Sigma-Aldrich). The biomass precursor chosen for this study was vegetable sponge (*Luffa cylindrica*), purchased from local vendors in the city of Vitória, Espírito Santo (Brazil). The crude oil (API 30.6) used in the preparation of the emulsions was sourced from the Espírito Santo basin (Brazil).

### 2.2 Synthesis of Cellulose Acetate

The biomass precursor was first cut into pieces approximately 3 × 5 cm^2^ in size, and the seeds were removed. The samples were then subjected to mechanical treatment using a Retsch SM300 knife mill with a 2 mm sieve. The chemical composition of the precursor, determined according to the procedure described by Morais et al.,^35^ was: 53 wt % cellulose, 20 wt % lignin and 27 wt % hemicellulose.

Cellulose extraction was performed using the organosolv method.^36^ The procedure consisted of three stages: initially, 30.70 g of the biomass precursor were subjected to reflux at 100 °C with water for 1 h. In the second stage, the water was replaced by 400 mL of a 20% v/v solution of HNO_3_ in 70% v/v ethanol and the mixture was refluxed for another 3 h. In the third step, the washed and filtered material was transferred to 400 mL of a 1 M NaOH solution, kept under magnetic agitation for 2 h at room temperature, and then washed until a neutral pH was achieved.

The synthesis of cellulose acetate was carried out using the homogeneous acetylation method.^36^ Initially, 10.00 g of the extracted cellulose were transferred to 200.00 mL of glacial acetic acid and the reaction mixture was magnetically stirred for 30 min at room temperature. Subsequently, a solution containing 0.80 mL of H_2_SO_4_ in 90.00 mL of glacial acetic acid was added to the mixture, which was then stirred for another 30 min. After this period, the solid was filtered and set aside. The resulting liquid was mixed with 320.00 mL of acetic anhydride and homogenized. The previously filtered solid was reintroduced into the reaction solution, which was kept under magnetic stirring for an additional 30 min. The system was then left to rest for 3 h. After this resting period, the system was stirred and distilled water was added to precipitate the cellulose acetate. The resulting material was neutralized with a 10% Na_2_CO_3_ solution until a neutral pH was reached.

### 2.3 Synthesis of GO and GO-TiO_2_

GO was prepared according to a modified Hummers’ method.^37^ Initially, 4.96 g of graphite were combined with 350.00 mL of 98% H_2_SO_4_. Simultaneously, 2.49 g of NaNO_3_ and 14.97 g of KMnO_4_ were slowly added to the mixture. This mixture was stirred in an ice bath for 2 h. After this period, 500.00 mL of distilled water were added and the mixture was stirred continuously for another 30 min. Then, approximately 50.00 mL of a 30% H_2_O_2_ solution were gradually added until the color of the mixture changed to brown, indicating the reduction of excess KMnO_4_. The resulting solid product was separated and washed by centrifugation, followed by freeze-drying. This material was then exfoliated using a Sonics VCX750 instrument, with a 13 mm ultrasonic tip, for 30 min, with a pulse cycle of 40/20 s, and at the maximum temperature of 50 °C. Chloroform was used as the exfoliation medium for the subsequent incorporation of GO into cellulose acetate and oleic acid was used as the exfoliation medium to obtain GO-TiO_2_.

Exfoliated GO in oleic acid was stored in a glovebox system (MBraun Purgebox) under a controlled atmosphere, where 100 μL of titanium butoxide (IV) were added. The mixture was heated by a microwave radiation synthesis system (CEM Discover SP) at 250 °C for 1 h.^38,39^ The resulting material was washed several times with acetone and chloroform by centrifugation and dried at room temperature; the obtained product was called GO-TiO_2_.

### 2.4 Preparation of Cellulose Acetate and Cellulose Acetate Membranes Incorporated with GO and GO-TiO_2._

The casting technique was employed to prepare the membranes. First, a previously dried 8% (m/v) cellulose acetate solution was prepared in chloroform and stirred for 24 h. This mixture was then evenly spread on a glass plate using a spreader to adjust the thickness to 0.025 mm, allowing the solvent to evaporate completely at 19 °C and 80% air humidity. Subsequently, the system was submerged in an ice water bath at 10 °C until the membrane detached from the glass. For the nanocomposite membranes, GO was incorporated after polymer dispersion. Previously exfoliated GO (in chloroform) was added to the polymeric dispersion at concentrations of 0.5 and 1% w/w, followed by magnetic stirring for 3 h. GO-TiO_2_ was dispersed in chloroform, subjected to an ultrasonic bath for 10 min, and then added to the polymeric dispersion at concentrations of 0.5 and 1% w/w, with subsequent magnetic stirring for 3 h. The membrane preparation process then followed the previously described steps. The prepared membranes were named as mCA (cellulose acetate membrane); mCA/GO_0.5% and mCA/GO_1% (cellulose acetate membranes modified with 0.5 and 1 wt % GO, respectively); mCA/GO-TiO_2__0.5% and mCA/GO-TiO_2__1% (cellulose acetate membranes modified with 0.5 and 1 wt % GO-TiO_2_, respectively).

### 2.5 Flux through Membranes in Water and Emulsion

**Figure 1:**
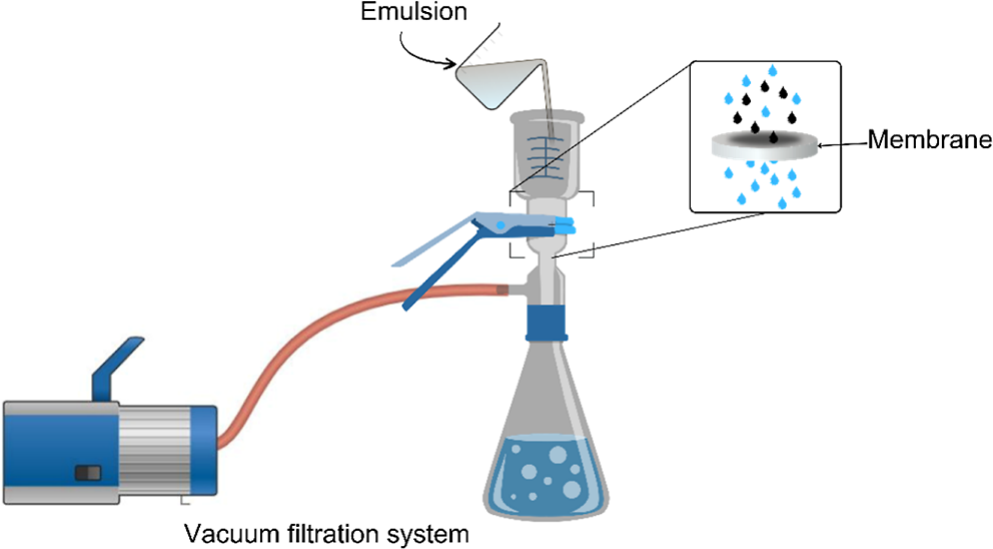
Schematic diagram of the experimental setup for the emulsion treatment.

The flux through the prepared membranes was investigated using both deionized water and oil-in-water emulsions. The emulsion was prepared by dispersing crude oil at a concentration of 200 ppm and 1% w/w surfactant (Triton-X100) in water using a high-speed agitator.^40^ Flux measurements were conducted using a vacuum filtration system, as show in Figure 1. The membranes were cut into discs with an effective area of 10.38 cm^2^ and placed with the top layer facing the water/emulsion surface. Filtration was performed under a pressure of 500 mmHg, and the time taken to collect every 50 mL of permeate up to a total of 150 mL was recorded. The water flux (*F*_w_) and emulsion flux (Fe) were calculated using eq 1.^23^
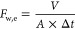
1

In this equation, *F*_w,e_ (Lm^–^^2^ h^–1^) is the volumetric flux, *V* (L) is the volume of collected permeate, *A* (m^2^) is the membrane’s effective area, and Δ*t* (h) is the filtration duration.

The emulsion and permeate concentrations were analyzed using a UV–Vis spectrophotometer (Global GTA-97).^41^ Standard solutions ranging from 5 to 200 ppm were prepared based on a 200 ppm crude oil solution in hexane. With absorbance readings taken between 200 and 400 nm, a calibration curve was generated using the highest absorbance peak at 259 nm. To extract crude oil from the emulsion, 50 mL of permeate was transferred to a settling funnel, followed by the addition of 2 drops of HCl and 25 mL of hexane. The mixture was manually shaken for 3 min and allowed to settle for 7 min to facilitate phase separation. The hexane-oil phase was then transferred to flasks containing anhydrous sodium sulfate to remove residual water. The samples were analyzed by UV–Vis spectrophotometry and the absorbance was measured at 259 nm to determine the crude oil concentrations in the emulsion and permeate.

The rejection efficiency of crude oil by the membrane (*R*,%) was calculated using eq 2,^42^ where *C*_0_ is the initial concentration of crude oil in the emulsion and *C* is the concentration of crude oil in the permeate.
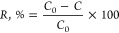
2

### 2.6 Characterization Techniques

Cellulose acetate was characterized based on its degree of substitution (DS).^43^ Initially, 0.10 g of cellulose acetate was added to a mixture containing 5.00 mL of standardized 0.25 M NaOH and 5.00 mL of EtOH. The system was left to stand for 24 h. After this period, 10.00 mL of standardized 0.25 M HCl solution were added and the mixture was allowed to stand for an additional period of 30 min. The excess HCl was titrated with a 0.25 M NaOH solution using phenolphthalein as an indicator. This procedure was conducted in triplicate, and based on these results, the degree of acetylation (% GA) was calculated using eq 3
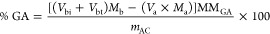
3

In this equation, % GA represents the percentage (w/w) of acetyl groups; *V*_bi_ is the initial NaOH volume; *V*_bt_ is the volume of NaOH used in titration; *M*_b_ is the molarity of NaOH obtained from standardization; *V*_a_ is the volume of HCl added; *M*_a_ is the molarity of HCl obtained from standardization; MM_GA_ is the molar mass of acetyl groups; and *m*_AC_ is the mass of acetate.

From eq 4, it is possible to calculate the DS value from the determined value of %GA^43^
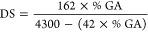
4

Cellulose acetate was also characterized for its viscometric molar mass (*M*_v_)^44^ using a Cannon-Fenske Schott 513 030C capillary viscometer (diameter Ø = 0.47 mm) at 25 °C. A dichloromethane/ethanol system (8/2 v/v) with a cellulose acetate concentration of 2 g/L was used. The *M*_v_ value was determined using the single point method, according to eq 5
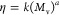
5Here, *k* and *a* are constants specific to the polymer–solvent pair at the chosen temperature. For the system at 25 °C, the values used in the present work were *k* = 13.9 × 10^–3^ mL.g^–1^ and *a* = 0.834.^44^

Fourier transform infrared (FTIR) spectroscopy analyses were performed using an Agilent Technologies Cary 630 ATR-FTIR spectrometer, which recorded 128 scans from 4000 to 650 cm^–1^ at a resolution of 4 cm^–1^.

Nuclear magnetic resonance (NMR) experiments were conducted on a 400 MHz Varian-Agilent spectrometer at 399.8 MHz for ^1^H and 100.5 MHz for ^13^C nuclei, corresponding to a magnetic field of 9.4 T. The solid-state ^13^C NMR spectra were recorded with the samples packed into 4 mm zirconia rotors, using either cross-polarization (CP) or direct polarization (DP); magic angle spinning (MAS) at 10 kHz and heteronuclear ^1^H decoupling was employed in both cases. The ^1^H–^13^C CP experiments were conducted with a π/2 pulse of 3.6 μs, a contact time of 1.0 ms, a recycle delay of 5.0 s, an acquisition time of 40.96 ms, and 800 scans. The quantitative ^13^C DP experiments were conducted for the cellulose acetate sample, using a π/2 pulse of 3.75 μs, a recycle delay of 200 s (long enough to avoid saturation issues), an acquisition time of 20.48 ms, and 544 scans. Additionally, solution ^13^C NMR experiments with ^1^H decoupling were conducted for the cellulose acetate sample, using a π/4 pulse of 4.45 μs, a recycle delay of 45 s, an acquisition time of 1.31 s, and 3000 scans; and solution ^1^H NMR experiments were conducted using a π/4 pulse of 6.15 μs, a recycle delay of 1 s, an acquisition time of 2.55 and 8 scans; for these experiments, a 50 mg sample was dissolved in 600 μL of dichloromethane-d_2_. In all cases, the NMR spectra were obtained by Fourier transform of the free induction decays and the chemical shifts were referenced to tetramethylsilane.

X-ray diffraction (XRD) experiments were performed on a Shimadzu XRD-6000 diffractometer using Cu Kα radiation (λ = 0.15418 nm) at 40 kV and 30 mA. The diffraction angle (2θ) ranged from 5 to 80° with a step size of 0.02° and a scanning speed of 2° per minute. The crystallinity index (CI) was calculated using eq 6(45)
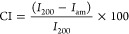
6

In this equation, *I*_200_ is the intensity of the peak located around 22–23° (associated with crystalline cellulose) and *I*_am_ is the intensity corresponding to the amorphous component, measured at 2θ ≈ 18–19°.

Raman spectra were recorded using a WITec alpha300R instrument with an excitation wavelength of 532 nm and a 100× objective lens. Thermogravimetry analysis (TGA) was conducted on a DTG-60H Shimadzu instrument in an oxidizing atmosphere; the samples were heated at a rate of 10 °C/min to 900 °C with a N_2_/O_2_ flow rate of 50 mL/min. The derivative thermogravimetry (DTG) curves were computed from the TGA data by numerical differentiation.

Scanning electron microscopy (SEM) images were obtained using a Jeol JSM-6610LV instrument at 20 kV with a resolution of 15 nm and magnification ranging from 500 to 10,000×. Energy-dispersive X-ray spectra (EDS) were recorded using a Bruker EDS detector coupled to the SEM, with an XFlash detector featuring an active area of 10 mm^2^ and an energy resolution of 121 eV. Optical microscopy (OM) images of the membranes were acquired using a Leica EZ4HD instrument. Transmission electron microscopy (TEM) images were recorded using a Jeol JEM1400 instrument, operated with an acceleration voltage of 120 kV. The powder samples were first dispersed in an ethanol solution, followed by stirring; a drop of the dispersion was then deposited onto a lacey support film in a Cu grid (Ted Pella no. 01885) and dried at room temperature, before being inserted in the sample holder to record the TEM images.

## 3 Results and Discussion

### 3.1 Characterization of the Biomass Precursor, Cellulose and Cellulose Acetate Samples

**Figure 2:**
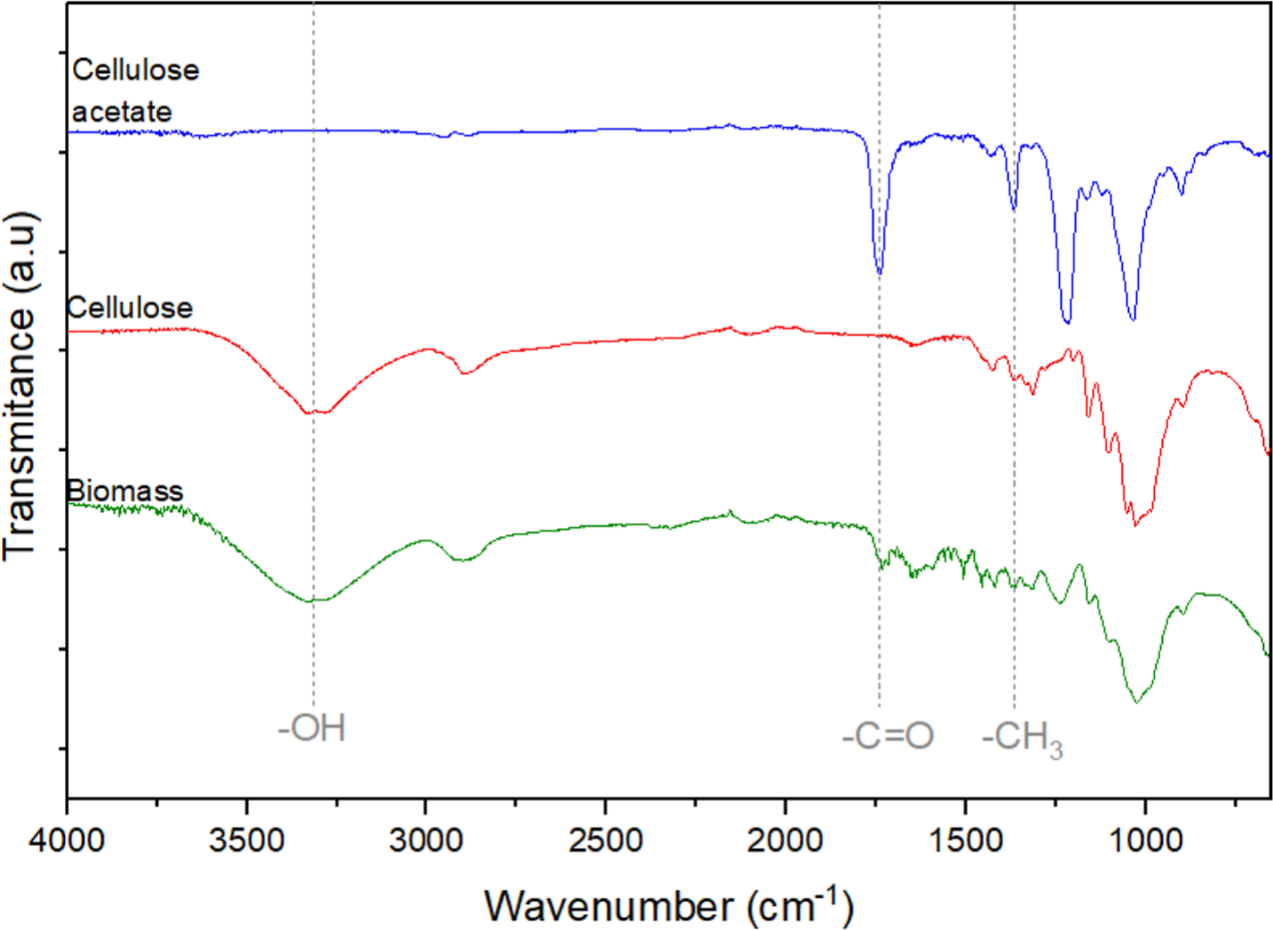
FTIR spectra of the biomass precursor, cellulose and cellulose acetate samples.

Figure 2 displays the FTIR spectra for the biomass precursor, extracted cellulose and cellulose acetate samples. The band observed at 1033 cm^–1^ in all three spectra corresponds to the C–O–C stretching vibrations inherent to the cellulosic chain.^46^ The band at 2897 cm^–1^ is attributed to CH_2_ and CH_3_ deformations present in both the biomass precursor and the cellulose fibers.^46^ In the spectrum of the biomass precursor, the band at 1732 cm^–1^ is assigned to the stretching mode of carbonyl groups present in hemicellulose,^46,47^ which is absent in the cellulose spectrum, indicating a reduction in the hemicellulose content of this sample. In the cellulose acetate spectrum, the band at 3311 cm^–1^, corresponding to intermolecular hydrogen bonds between the cellulose hydroxyl groups, is absent. The increased intensities of the bands at 1366 and 1740 cm^–1^ indicate characteristic CH_3_ deformations of acetyl groups and carbonyl stretching of acetyl groups, respectively;^48^ these changes confirm the substitution of hydroxyl groups by acetyl groups in the prepared cellulose acetate sample.

**Figure 3:**
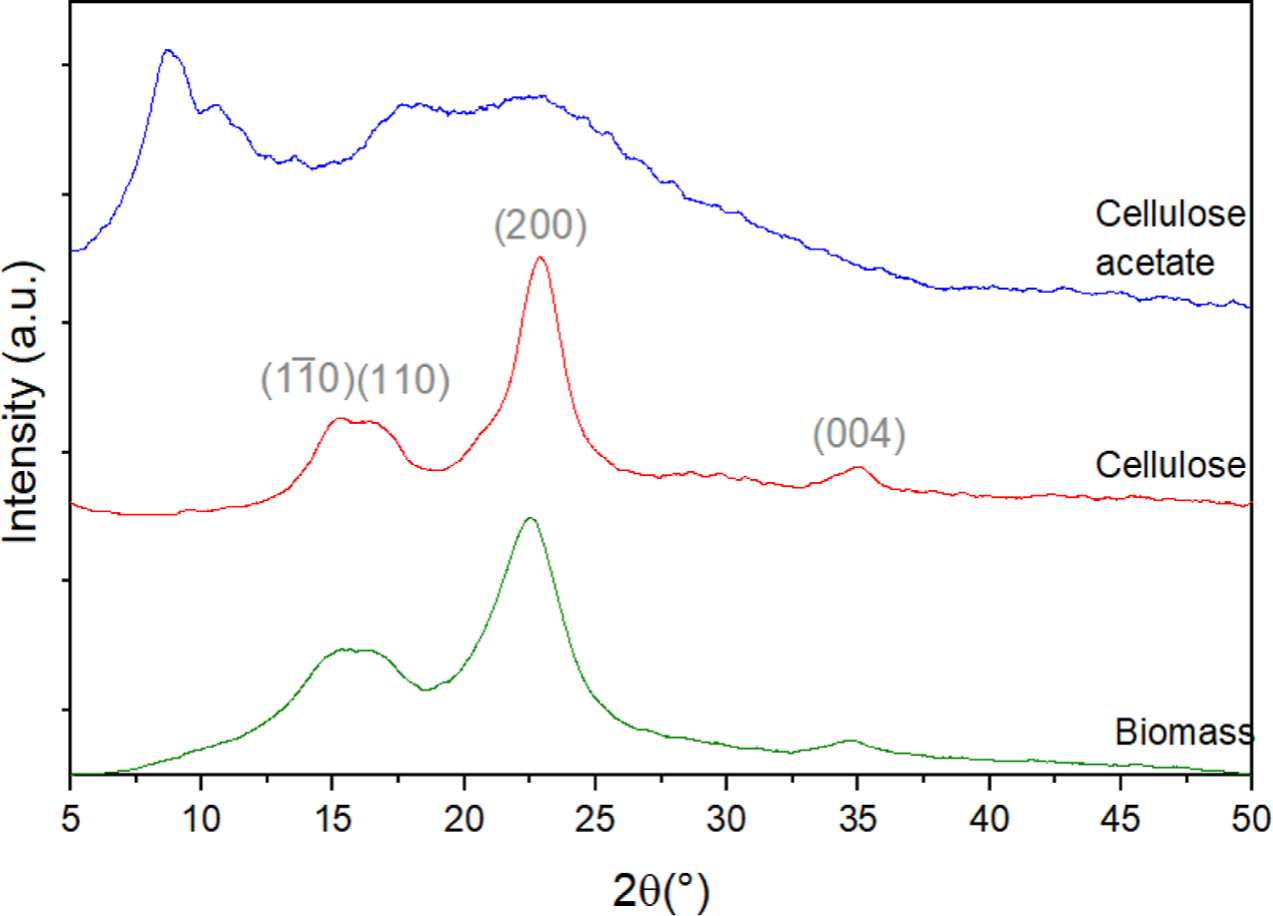
X-ray diffractograms of the biomass precursor, cellulose and cellulose acetate samples.

Figure 3 shows the X-ray diffractograms of the biomass precursor, cellulose and cellulose acetate. The peaks observed at 2θ = 15, 16, 23 and 35° are characteristic of native cellulose (PDF card no. 50-2241).^49^ These diffraction peaks are typical of the crystalline structure of cellulose observed in both biomass and cellulose samples.^48^ The CI was 67% for the biomass precursor and 83% for the cellulose sample, indicating that the extraction process reduced the fraction corresponding to the amorphous part, resulting in a more crystalline cellulosic material. Cellulose can exist in different crystalline phases, with the most common being referred to as phases Iα and Iβ; phase Iα has a triclinic unit cell, while phase Iβ has a monoclinic unit cell.^50,51^ The predominance of each polymorph varies depending on the cellulose source, with I_β_ being more prevalent in plants.^52^ The X-ray diffractograms of both the biomass precursor and the cellulose sample display the characteristic (11̅0), (110), (200) and (004) planes^43^ of the I_β_ phase of cellulose.^45^ For the cellulose acetate sample, the X-ray diffractogram indicates a change in material crystallinity, notably marked by the development of a peak at 2θ = 9° and a broadening of the diffraction peaks. The broad peaks that occur at diffraction angles of 2θ = 9, 18 and 23° are characteristic of cellulose triacetate.^53^ Acetylation reduces the number of hydrogen bonding interactions that organize the cellulose chains, increasing the spacing between the fibers in comparison with cellulose. Consequently, the chains in cellulose triacetate are considerably less organized than the cellulose chains.^53,54^

**Figure 4:**
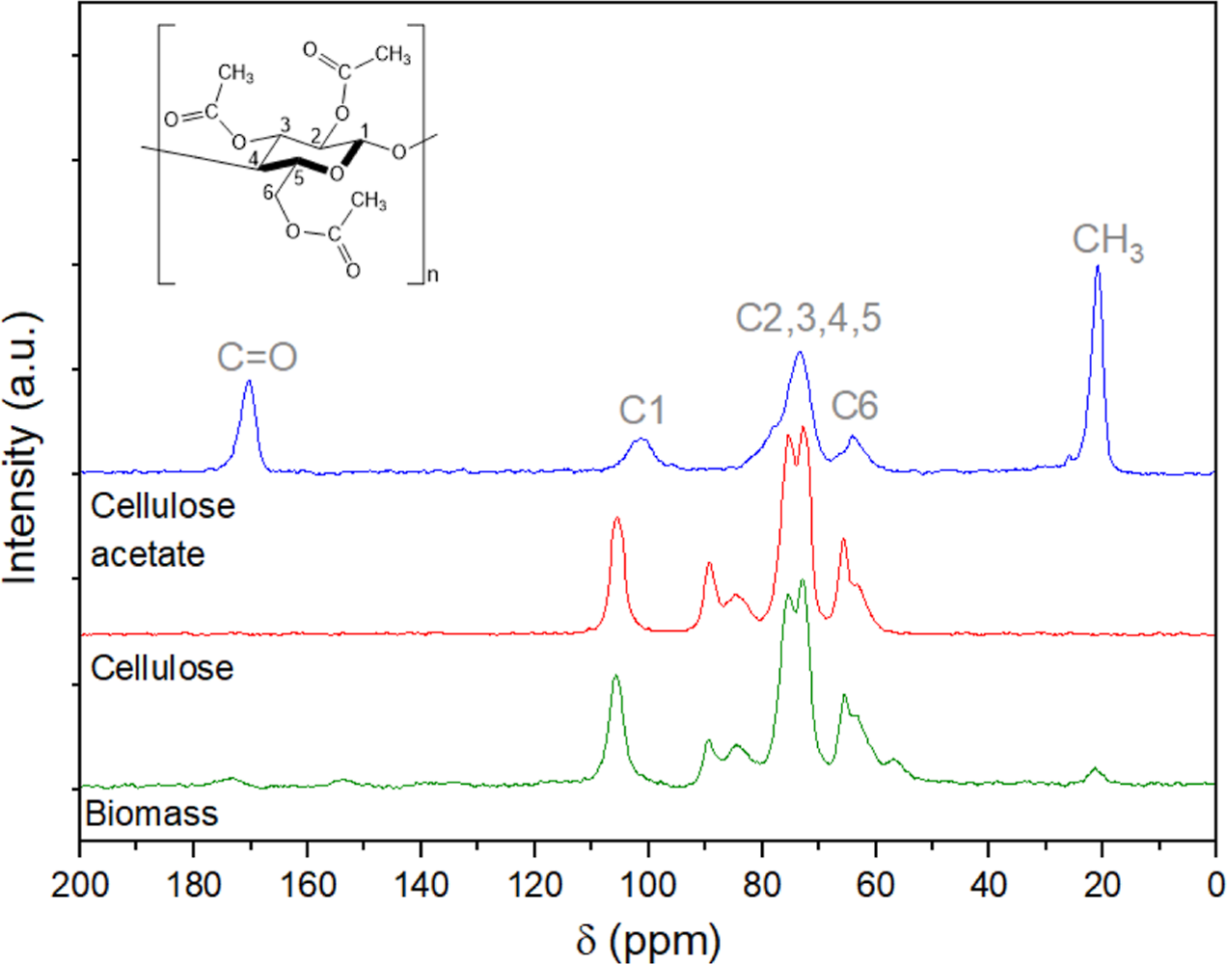
^13^C CP/MAS NMR spectra of the biomass precursor, cellulose and cellulose acetate samples.

The solid-state ^13^C NMR spectra obtained for the biomass precursor, cellulose, and cellulose acetate samples are shown in Figure 4. In the spectrum of the biomass precursor, the signals at 174 and 21.5 ppm correspond to the acetyl groups of hemicellulose. Signals between 110 and 150 ppm (^13^C nuclei in aromatic carbon atoms) and at 56 ppm (methoxyl groups) are attributed to lignin.^48,55^ These signals from hemicellulose and lignin are absent in the cellulose spectrum, indicating its high purity. In the cellulose spectrum, signals corresponding to different carbon atoms in the monomeric anhydroglucose unit were observed: C1:105 ppm; C2, C3, C5:73–75 ppm; C4:84 and 89 ppm; and C6:63 and 66 ppm.^36,56^ The presence of narrower and stronger peaks at 89 and 66 ppm in the cellulose spectrum also indicate that the cellulose extraction method resulted in a more crystalline form compared to the precursor,^36,57^ which is consistent with the XRD findings discussed above. In the cellulose acetate spectrum, distinct and intense signals appear at 170 and 21 ppm, attributed, respectively, to carbonyl and methyl groups,^36^ confirming the successful acetylation of cellulose. Additional spectral changes associated with the structural modifications in cellulose after acetylation also include: the changes of chemical shifts of the peaks due to C1 and C4 carbons; the disappearance of the doublet corresponding to carbons C2, C3, and C5; the disappearance of the doublet corresponding to the C6 carbon; and the broadening of all the detected peaks. These changes reflect alterations in the cellulose structure following acetylation, pointing to a reduction in the crystallinity of the material, once more in agreement with the above-discussed XRD results.^48,58^

The CP method used to record the ^13^C NMR spectra shown in Figure 4 allows the achievement of spectra with good signal/noise ratio; however, the relative intensities of the several peaks present in these spectra do not reflect the distribution of the amounts corresponding to each identified chemical group, since the efficiency of the CP process depends on the strength of the ^1^H–^13^C dipolar coupling.^48,55^ Therefore, quantitative ^13^C DP/MAS and solution ^1^H and ^13^C NMR spectra were also recorded for the cellulose acetate sample (Figure S1 Supporting Information). By comparing the intensities of the peaks due to the acetyl groups with those of the anhydroglucose unit in the ^1^H NMR spectrum,^59^ the DS was estimated to be around 2.9, suggesting the formation of cellulose triacetate. This finding is consistent with the results obtained by the chemical route described in Section 2.6, which indicated that 41.57 ± 0.59% of the hydroxyl groups in cellulose were acetylated, leading to a DS value of 2.64 ± 0.06. These results confirm thus the formation of cellulose triacetate, corroborating the above-discussed FTIR, XRD, and NMR findings. The viscometric molar mass of the produced cellulose acetate was determined to be 38,722 g·mol^–1^. Cellulose acetates with similar molar masses have previously been used for membrane production, as documented in the literature.^23,60^

**Figure 5:**
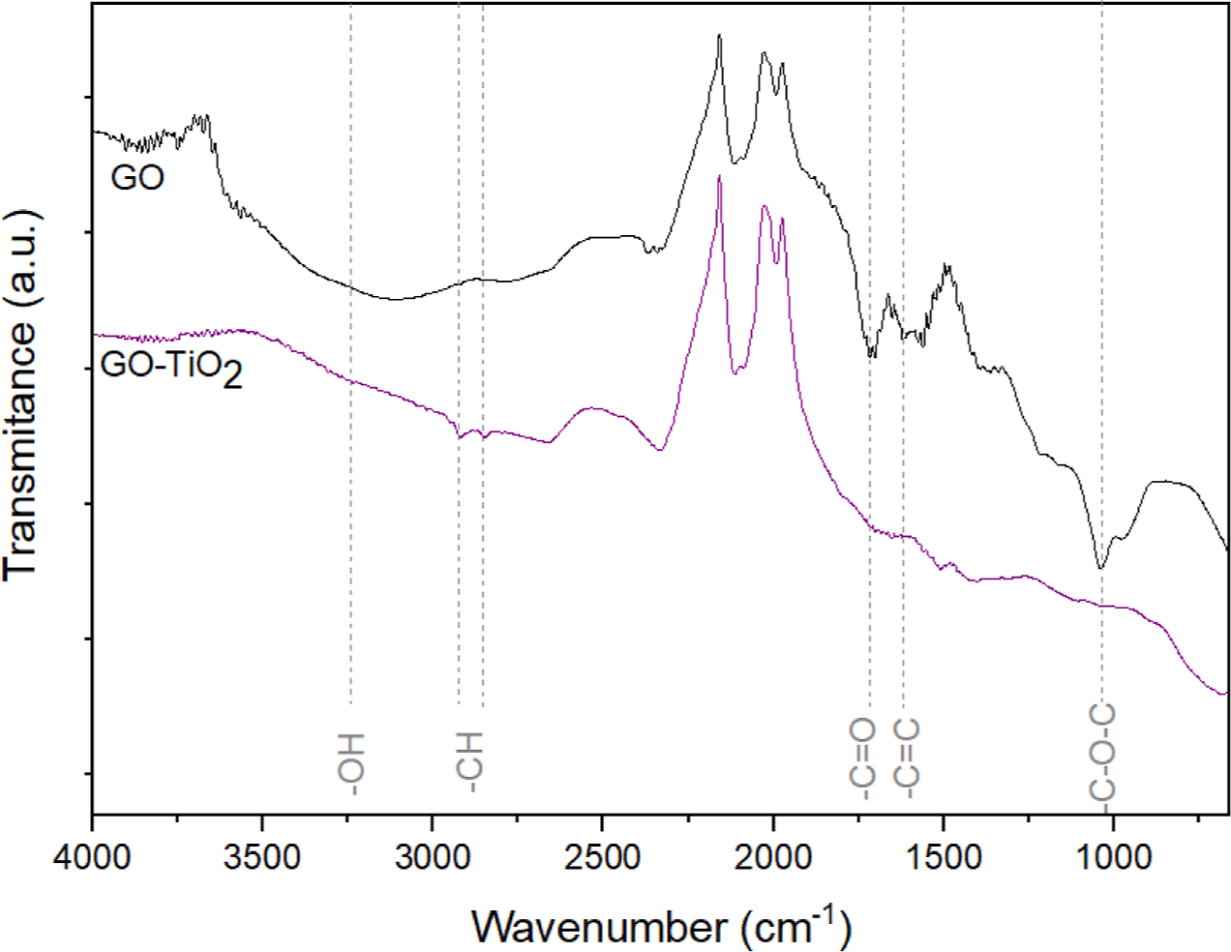
FTIR spectra of GO and GO-TiO_2_.

### 3.2 Characterization of GO and GO-TiO_2_

Figure 5 displays the FTIR spectra obtained for the GO and GO-TiO_2_ samples. The bands observed at 1033, 1613, and 1710 cm^–1^ are typical of chemical groups present in GO and are attributed to stretching of epoxy, C=C, and carboxylic groups, respectively.^61,62^ The band in the region of 3200 cm^–1^ is associated with hydroxyl group stretching vibrations.^61,62^ The observation of these bands indicates then the successful preparation of GO by the modified Hummers’ method. Compared with the GO spectrum, the GO-TiO_2_ spectrum shows additional bands at 2922 and 2854 cm^–1^, characteristic of alkyl groups from oleic acid used in the synthesis of the TiO_2_ nanoparticles,^34^ and at approximately 700 cm^–1^, indicative of Ti–O–Ti bond stretching vibrations.^63,64^ The decrease in the intensity of the bands due to oxygen-containing groups in the GO-TiO_2_ spectrum in comparison with the GO spectrum suggests the reduction of GO to rGO^60,62^ with increasing temperature during TiO_2_ nanoparticle formation.^63,65^

**Figure 6:**
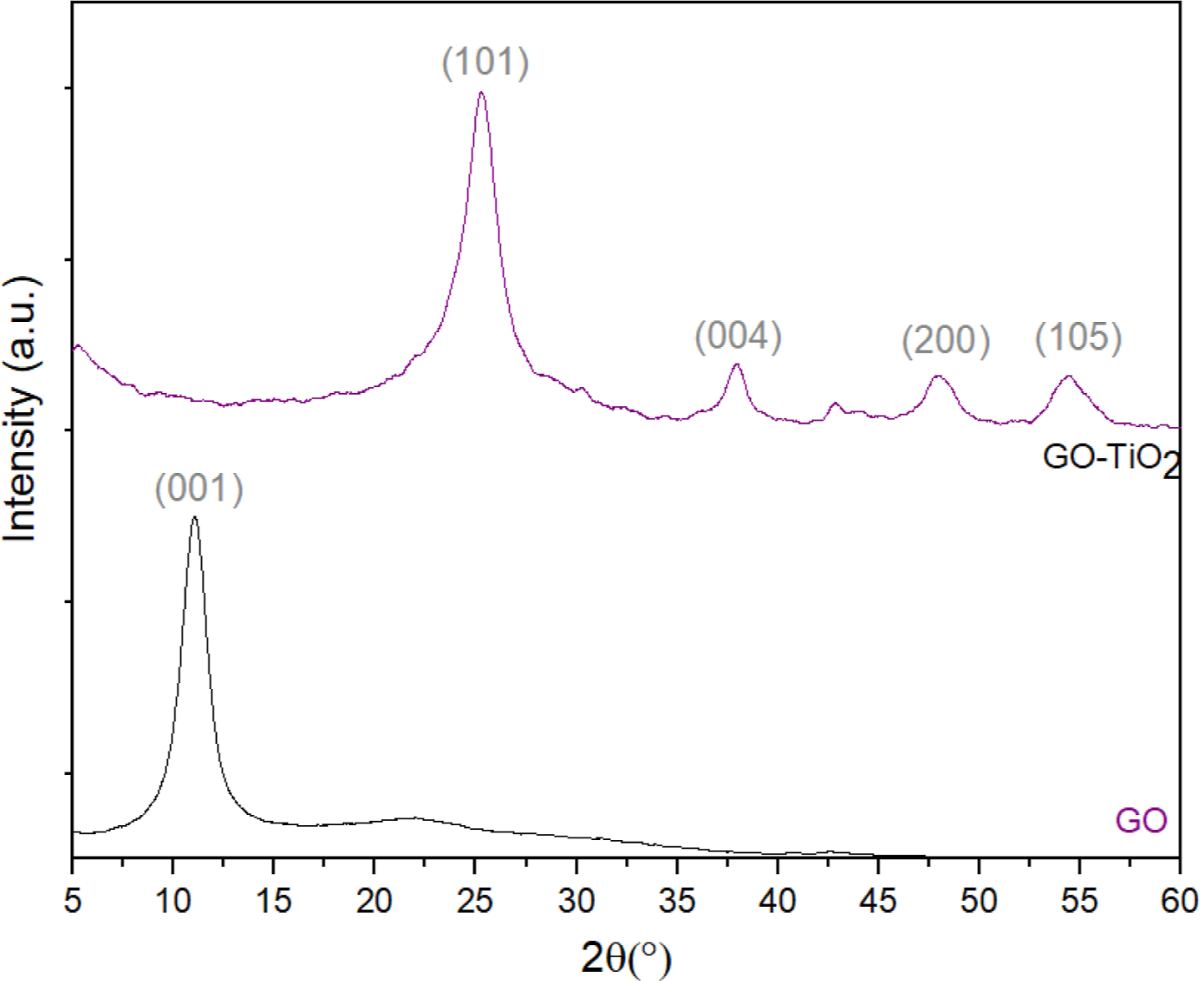
X-ray diffractograms of GO and GO-TiO_2._.

In the X-ray diffractogram of GO shown in Figure 6, the intense diffraction peak at 2θ = 11° is associated with the (001) planes of the GO structure.^66^ The interlayer spacing calculated by Bragg’s Law^67^ for the (001) planes of GO was determined to be 0.80 nm, which is much larger than the value corresponding to hexagonal graphite (0.335 nm)^66−68^ This increase in the interlayer spacing is a consequence of the oxidation process during GO production, with the introduction of oxygen-containing groups and water molecules between adjacent layers in the GO structure.^69^ In the X-ray diffractogram obtained for GO-TiO_2_, peaks are observed at 25° (101), 38° (004), 48° (200) and 54° (105), characteristic of the anatase crystal phase of TiO_2_ (PDF card no. 21-1272).^49^ Notably, the diffraction peak previously present at 11° for GO is not present in the GO-TiO_2_ XRD pattern, indicating its reduction (i.e., rGO formation) due to the heat treatment used for TiO_2_ deposition,^68^ whereas the (002) peak of rGO overlaps with the TiO_2_ peak at 25°. Using the Scherrer equation,^34^ the average crystallite size of TiO_2_ nanocrystals was estimated at 5.8 ± 0.3 nm.

**Figure 7:**
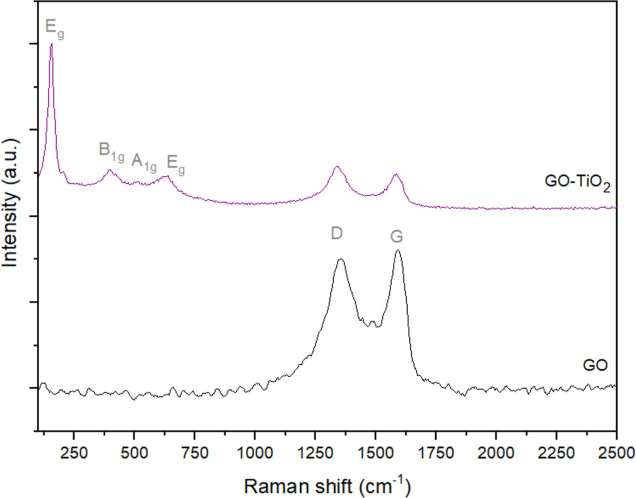
Raman spectra of GO and GO-TiO_2_.

The Raman spectra of both the GO and GO-TiO_2_ samples are presented in Figure 7. In both spectra, the peaks at 1337 and 1588 cm^–1^ correspond to the D and G bands commonly found in Raman spectra of graphenic materials, respectively.^70^ The G-band is typical of sp^2^ carbons present in GO and rGO, while the D-band is attributed to structural defects and sp^3^ carbons formed as a consequence of the oxidation process.^71^ Additional bands observed in the spectrum of GO-TiO_2_ include those of *E*_g_ (151 and 637 cm^–1^), *B*_1g_ (393 cm^–1^), and *A*_1g_ (511 cm^–1^) modes, which are characteristic of the anatase crystal phase of TiO_2_,^72^ corroborating the above-discussed XRD results.

**Figure 8:**
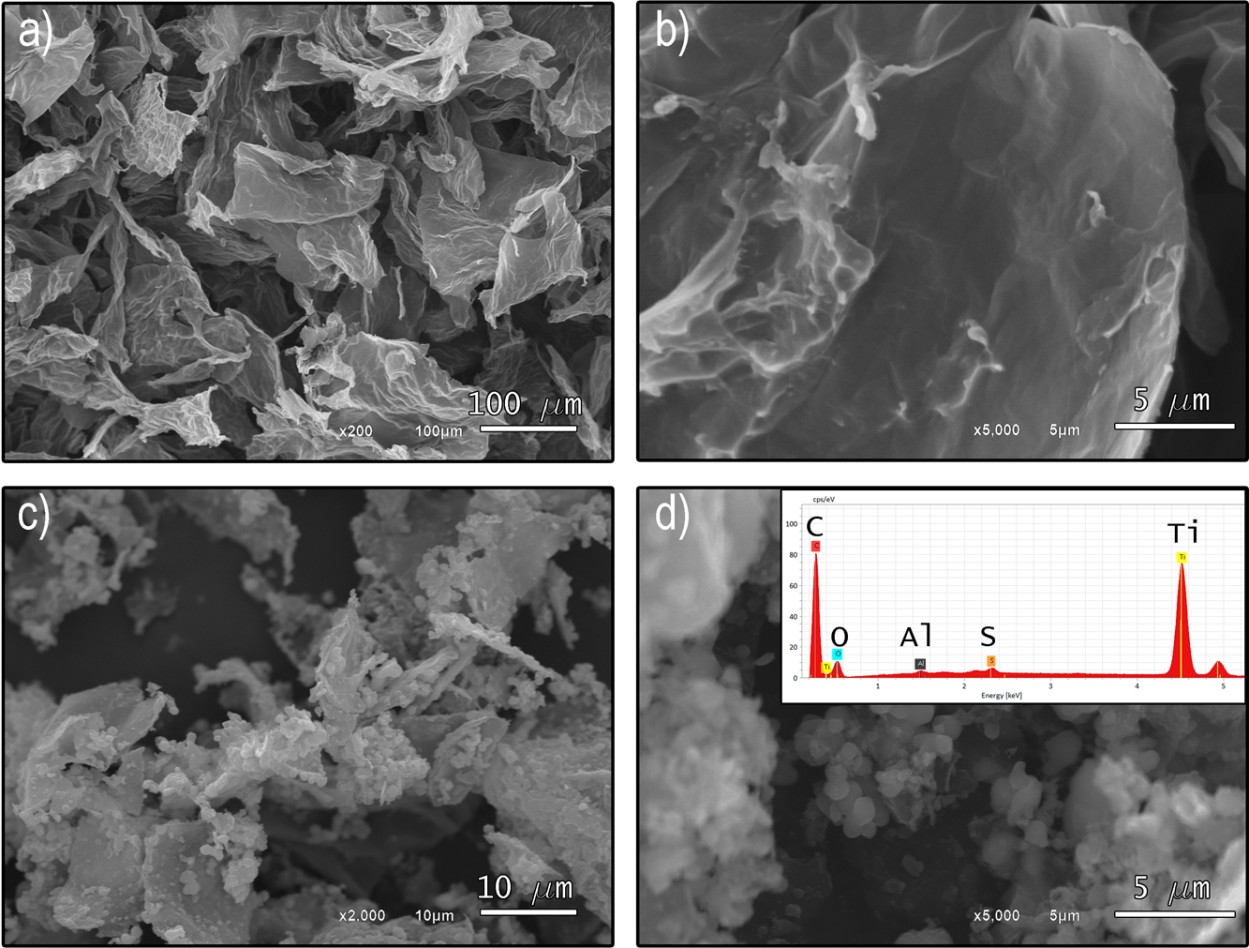
SEM images of GO at 200× (a), GO at 5000× (b), GO-TiO_2_ at 2000× (c) and GO-TiO_2_ at 5000× (d) magnification. The inset in (d) presents the microanalysis characterization by EDS of GO-TiO_2_.

Figure 8 shows the SEM images of the GO and GO-TiO_2_ samples. In images (a) and (b), GO sheets are depicted in a wrinkled and randomly arranged way, as it is typical of highly oxidized GO samples.^61,67^ This arrangement is a result of the oxidation process affecting the graphene sheets. In images (c) and (d), the SEM images show GO sheets functionalized with TiO_2_ nanoparticles. Compared to those of GO, these sheets appear more dispersed and smaller. This dispersion is attributed to the physical exfoliation process of GO during functionalization with TiO_2_ nanoparticles. It is also possible to observe the TiO_2_ nanoparticles deposited on the GO sheets, indicating successful functionalization with TiO_2_. This point is supported by studies reported by Ye et al.^63^ and Yue et al.,^42^ who provided similar SEM images and findings. In the inset of Figure 8d, the EDS spectrum reveals the presence of C, O, and Ti as the main elements in the GO-TiO_2_ sample. The presence of Ti confirms the successful incorporation of TiO_2_ in the material. The Al peak originates from the sample holder, while the S signal is due to the presence of residual S-containing groups in the GO sample that was prepared by the oxidation of graphite using the Hummers’ method (which involves the use of large amounts of H_2_SO_4_).^61,67^

**Figure 9:**
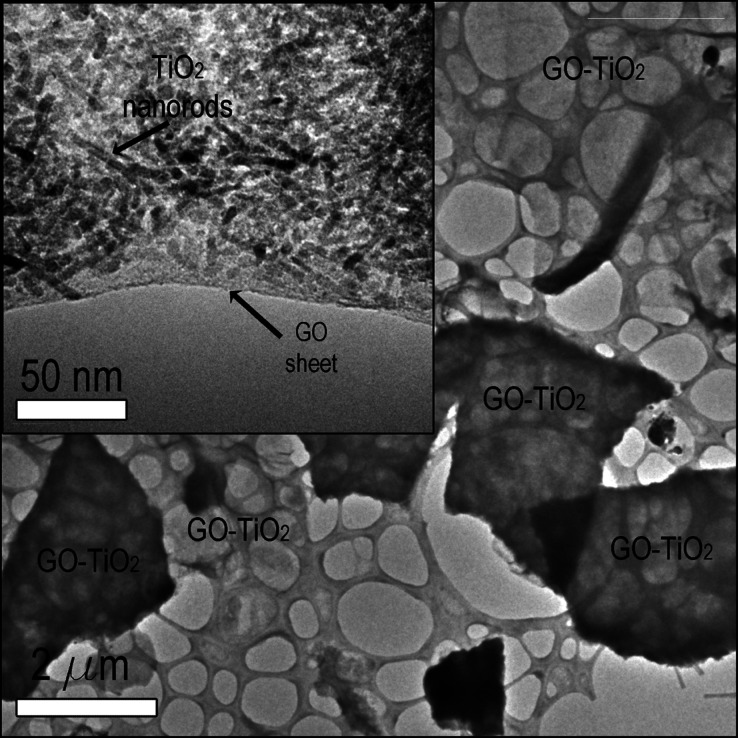
TEM images of GO sheets functionalized with TiO_2_ nanostructures. The inset shows a high-magnification view, highlighting the presence of TiO_2_ nanorods uniformly distributed on the surface of the GO sheets.

The TEM images presented in Figure 9 provide a detailed structural characterization of the GO-TiO_2_ sample. The lower magnification image provides a general view, indicating the microscale distribution of functionalized GO sheets. The inset, with higher magnification, reveals the deposition of TiO_2_ nanorods on the GO sheets, indicating effective functionalization of the GO surface with TiO_2_. TGA was used as an additional technique for comparing the thermal behavior of GO and GO-TiO_2_.^73^ The TGA curve of the GO sample, shown in Figure S2a (Supporting Information), exhibits a weight loss starting at ca. 250 °C, due to the elimination of oxygenated functional groups present in the GO structure; at approximately 520 °C, the last weight loss is due to the oxidation of the remaining carbon-rich material.^74^ The TGA results indicate an ash content of 33% for the GO-TiO_2_ sample, which is attributed to the presence of the TiO_2_ nanoparticles in the composite material.^75^ This functionalization confirmed the successful incorporation of TiO_2_ on the GO sheets, corroborating the observations made in connection with the discussion of the SEM/TEM images and the XRD analysis.

### 3.3 Characterization of the Membranes

**Figure 10:**
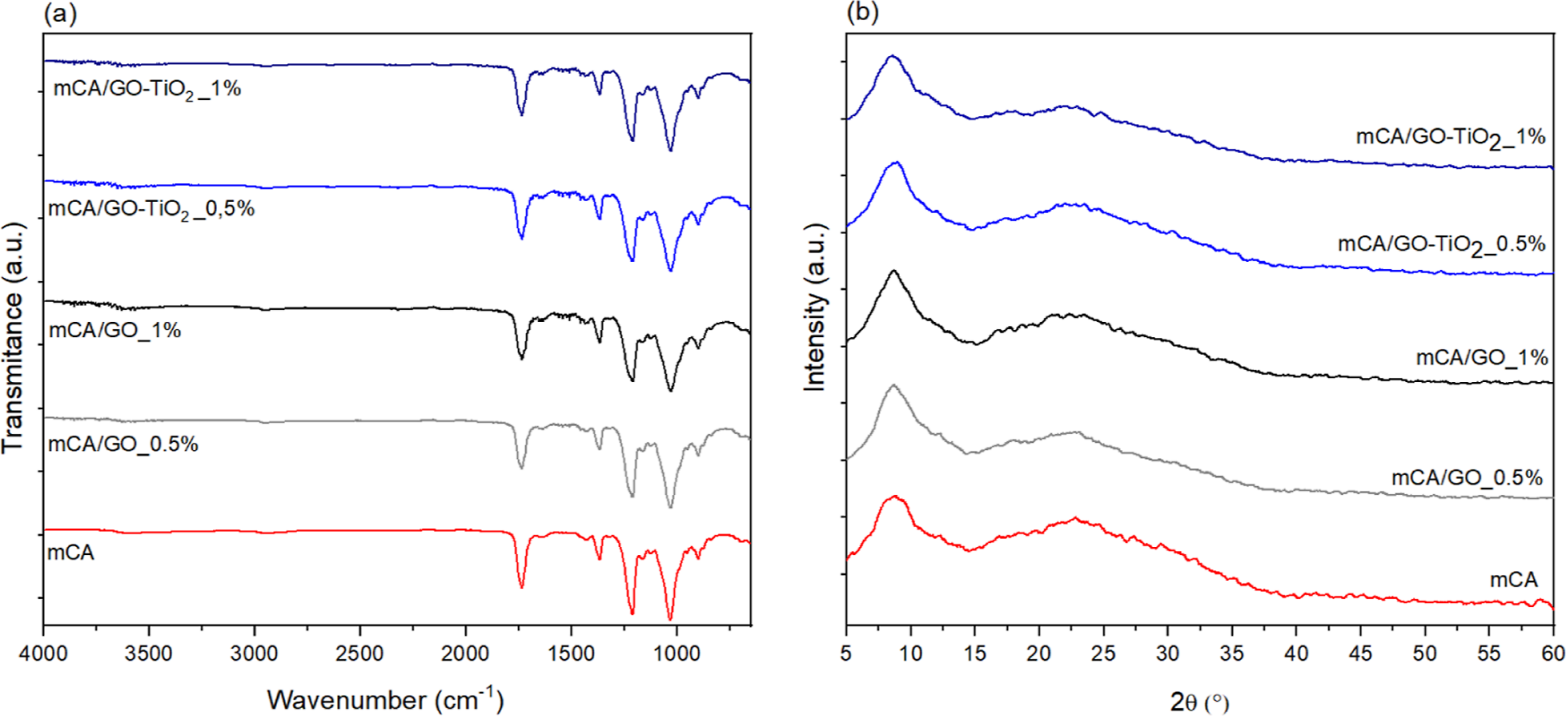
(a) FTIR spectra and (b) XRD patterns of the mCA, mCA/GO_0.5%, mCA/GO_1%, mCA/GO-TiO_2__0.5% and mCA/GO-TiO_2__1% membranes.

The FTIR spectrum of the membranes, shown in Figure 10a, displayed the characteristic bands of cellulose acetate, as previously discussed in Section 3.1. No distinct bands associated with the incorporated materials (GO and GO-TiO_2_) were observed. This is likely due to the reduced fraction of these materials in the membranes and the dominant signals from the functional groups present in the cellulose acetate. Similarly, this observation explains why the XRD patterns shown in Figure 10b are dominated by the diffraction peaks due to cellulose acetate. At the given concentrations, the incorporated materials did not significantly impact the crystal structure of the cellulose acetate. The membrane fabrication process induces a preferential orientation for the cellulose acetate chains, which explains the increase in the relative intensity of the diffraction peak at 9° in the XRD patterns of the membranes in comparison with the X-ray diffractogram of cellulose acetate powder (Figure 3).

**Figure 11:**
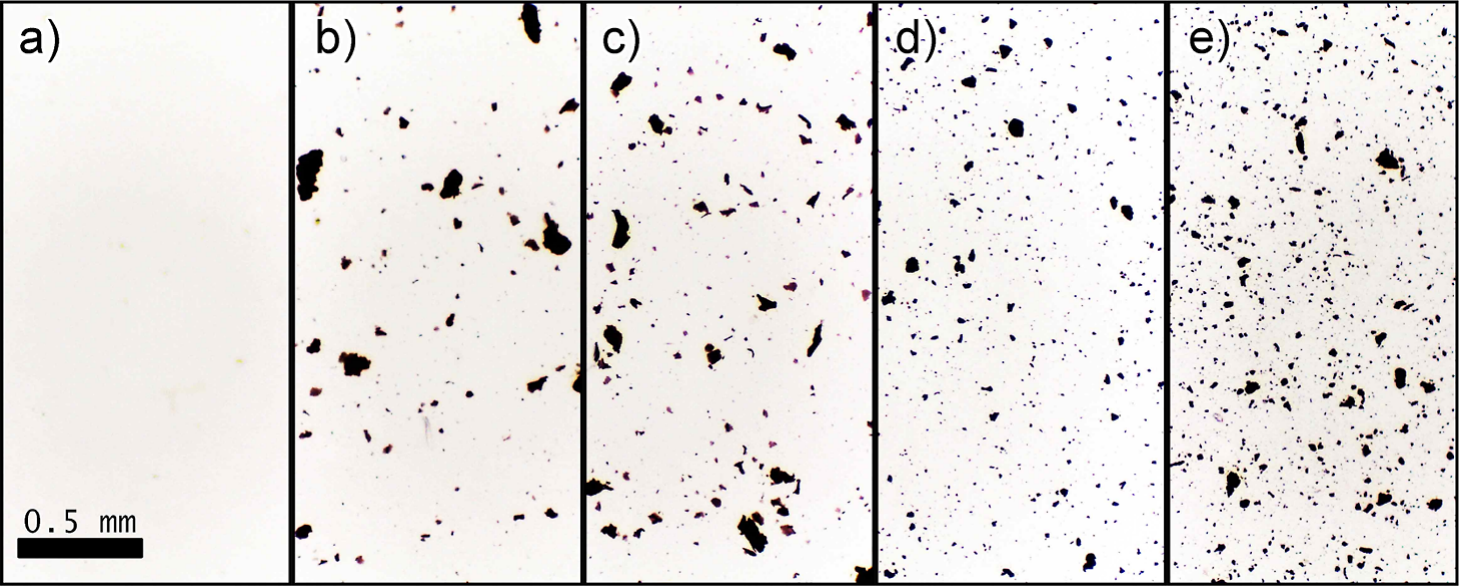
OM images of the membranes (a) mCA, (b) mCA/GO_0.5%, (c) mCA/GO_1%, (d) mCA/GO-TiO_2__0.5% and (e) mCA/GO-TiO_2__1%. All images were taken at the same magnification; the scale bar presented in (a).

The OM images of the prepared membranes confirmed the incorporation of GO and GO-TiO_2_ into the cellulose acetate membranes. Figure 11b,c show larger aggregates of particles present in the GO-modified membranes. This segregation is attributed to the hydrophilic nature of GO, which is not completely compatible with cellulose acetate^28,30^ and has also low compatibility with chloroform,^76^ the solvent used in the preparation of membranes. In contrast, the membranes incorporated with GO-TiO_2_, as shown in Figure 11d,e, exhibit significantly more dispersed particles of smaller sizes. This indicates that the functionalization of GO with TiO_2_ through the solvothermal process enhances the interaction with cellulose acetate, promoting better dispersion in the matrix.^33,34^ This improved dispersion is due to the modification of the surface properties of GO by TiO_2_ and oleic acid, which increases the compatibility of GO with both the polymer matrix and the used solvent.

**Figure 12:**
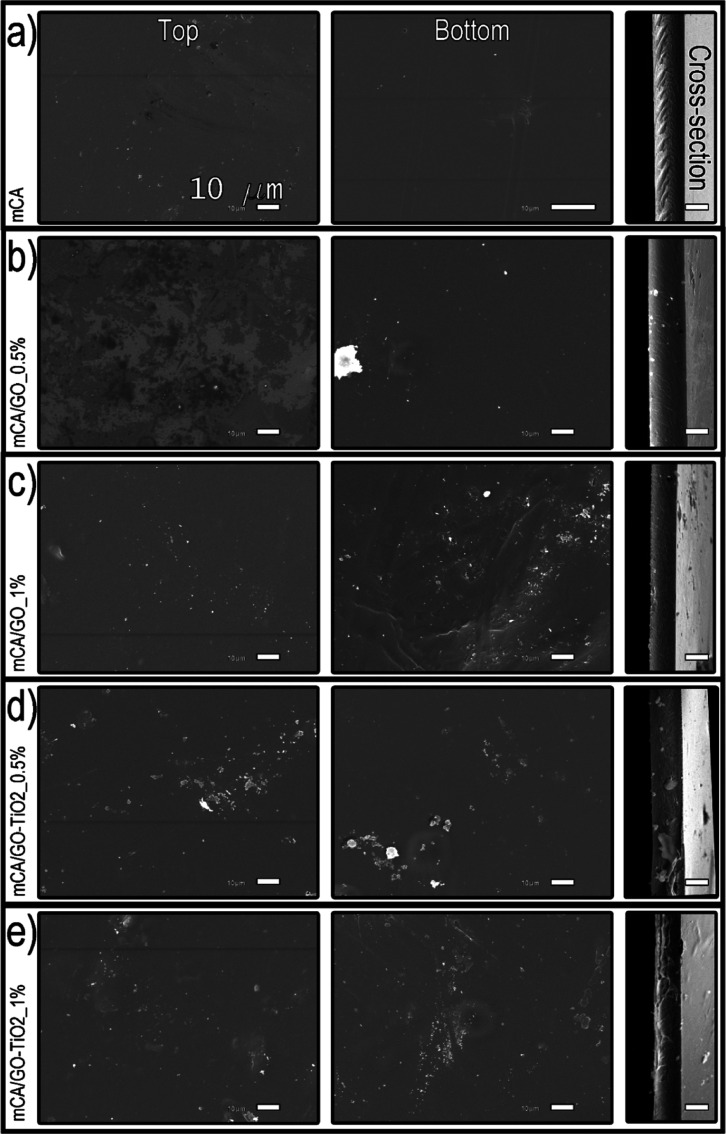
SEM images of the top surface, bottom surface, and cross section of the prepared membranes: (a) mCA, (b) mCA/GO_0.5%, (c) mCA/GO_1%, (d) mCA/GO-TiO_2__0.5% and (e) mCA/GO-TiO_2__1%. All scale bars represent 10 μm.

The membrane morphology was also evaluated using SEM; the recorded images are shown in Figure 12. Surface images revealed that all the membranes had smooth surfaces, with no pores on either side. Fracture images further confirmed the absence of pores throughout the cross-section perpendicular to the plane of the membrane. Therefore, the membranes produced can be classified as dense membranes.^77−79^

In the membranes containing 1% of the incorporated materials, a larger number of particles (visible as clear regions in the images) is observed on the lower surface, indicating the deposition of GO and GO-TiO_2_ during the membrane formation process. In contrast, the membranes containing 0.5% of GO and GO-TiO_2_ showed better dispersion of the incorporated materials on both surfaces. Furthermore, the GO and GO-TiO_2_ particles can be observed throughout the membrane cross sections.

### 3.4 Membrane Flux and Rejection Tests

Figure S3 (Supporting Information) shows that, in the pure water flux assay, the flux through the mCA membrane decreased due to compaction during the experiment. For the mCA/GO_0.5% membrane, the water flux remained constant and was similar to that of the mCA membrane, indicating that the addition of 0.5% GO has not significantly changed the membrane’s structure for water flux.^80^ In the case of the mCA/GO_1% membrane, the water flux also remained constant but was reduced by half compared to that of the mCA membrane. According to Ghaseminezhad, Barikani and Salehirad,^81^ the addition of 1% GO particles to porous cellulose acetate membranes led to increased hydrophilicity but also caused a decrease in flux due to the reduction of macrovoids occupied by the particles. In this study, which involved dense membranes, the decrease in flux with 1% GO incorporation could be attributed to the deposition of these particles on the lower surface of the membrane, as observed in the SEM images. This deposition occurs due to the formation of aggregates and the low dispersion of the GO particles in the polymer matrix. For the mCA/GO-TiO_2__0.5% and mCA/GO-TiO_2__1%, an even larger flux reduction is observed compared to that of the pure membrane. In these cases, the particles are better dispersed in the polymer matrix and the functionalization with oleic acid also changed the hydrophilicity of the membranes. No compaction effect was observed in the incorporated membranes throughout the experiment, as shown in Figure S3.

**Figure 13:**
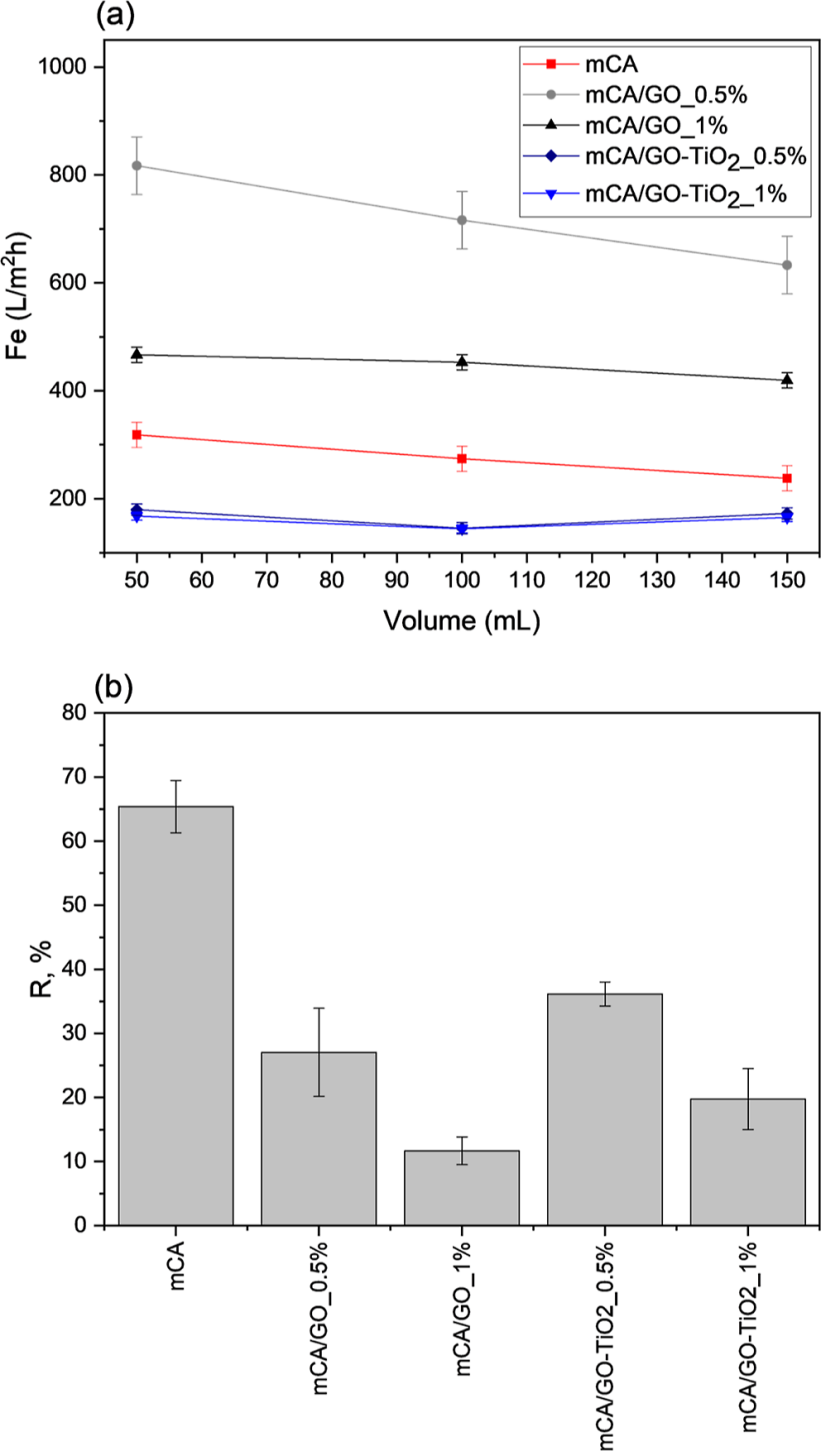
(a) Emulsion flux (F_e_) through the prepared membranes, plotted as a function of the filtrate volume. (b) Oil rejection determined for each membrane.

The mCA membrane exhibited an intermediate flux during the emulsion separation process compared to that of the incorporated membranes, as shown in Figure 13. A reduction in flux during the filtration process was observed, which is associated with the accumulation of crude oil on the membrane surface.^82^ Regarding selectivity, the mCA membrane showed a rejection rate of approximately 60% (Figure 13b), indicating the efficiency of the pure polymer matrix in removing crude oil from the emulsion. Shoba and Jeyanthi^83^ reported good oil-in-water separation efficiency for porous cellulose acetate membranes prepared under different conditions, noting the need for characteristic control to minimize fouling.

Compared with the mCA membrane, the mCA/GO_0.5% membrane showed a larger emulsion flux. The use of GO at this concentration resulted in larger incorporation into the polymer matrix, providing additional channels for permeate flux. This increased flux indicates the efficacy of adding GO at optimized concentrations. For this membrane, a decrease in flux during the filtration process can be attributed to the accumulation of crude oil on its surface.

A higher flux in comparison with the mCA membrane was also observed for the mCA/GO_1% membrane. The effect of oil accumulation on the surface, which typically causes flux reduction, is compensated by the formation of regions where the emulsion passage is easier, due to the detachment of GO particles. This keeps the flux during filtration test but negatively impacts the selectivity. The detachment of GO particles was observed during the emulsion filtration experiment only for the mCA/GO_1% membrane. The SEM images shown in Figure 12 revealed that the GO particles were not well dispersed in the polymer matrix in this case. Ghaseminezhad, Barikani and Salehirad^81^ reported good properties for cellulose acetate membranes incorporated with up to 1% GO, noting that higher amounts led to the formation of agglomerates that decreased interactions with the polymer matrix. In this work, at 1% GO, the formed agglomerates reduced the surface area of the particles and consequently their interaction with the polymer matrix, facilitating their detachment during the filtration experiment. Despite exhibiting higher flux values, the selectivity of the mCA-GO membranes was inferior in comparison with the mCA membrane (Figure 13b). In particular, the mCA/GO_1% membrane showed the lowest rejection rate, which is attributed to the facilitated passage of the emulsion through the regions where the detachment of the GO particles occurred.

The emulsion flux values through the membranes modified with GO-TiO_2_ were lower than those of both the mCA and the mCA-GO membranes (Figure 13a). The lower average flux is attributed to the functionalization of TiO_2_ with oleic acid, which decreases hydrophilicity and promotes better dispersion in the polymer matrix. The lower affinity of the additive for water led to reduced flux. The similar emulsion flux values observed for mCA/GO-TiO_2__0.5% and mCA/GO-TiO_2__1% membranes can be attributed to saturation effect in nanoparticle dispersion, where higher concentrations can lead to nanoparticle aggregation.^84^ This aggregation can impair the homogeneity of the polymer matrix and affect the efficiency of GO-TiO_2_ dispersion, resulting in a reduced active area available for flux.^85,86^ On the other hand, these membranes demonstrated superior rejection compared to that of the mCA-GO membranes due to the better compatibility of the additive with the matrix, which preserves the membrane structure and promotes greater selectivity. A two-way ANOVA statistical analysis was conducted, revealing that the membrane composition significantly impacts the emulsion flux. The analysis at a significant level 0.05 in a Tukey test indicated that the mCA, mCA/GO-TiO_2__0.5% and mCA/GO-TiO_2__1% samples are statistically similar, while the other membranes show significant differences.

The results obtained in the emulsion separation tests indicate that the pure cellulose acetate membrane offers satisfactory performance in terms of crude oil rejection but is limited by fouling problems. The addition of 0.5% GO significantly improves permeate flux but causes the accumulation of crude oil on its surface. Membranes with 1% GO also improve permeate flux, but the selectivity is compromised due to the detachment of GO particles, which leads to the formation of regions that facilitate the emulsion flux with low selectivity. Despite exhibiting a lower fluw, the membranes modified with GO-TiO_2_ did not show fouling issues and offered superior rejection compared to the membranes modified just with GO. The results suggest that the incorporation of additives such as GO and GO-TiO_2_ may be an effective strategy for optimizing the performance of cellulose acetate membranes in petroleum separation applications, with a careful balance between flux and selectivity required to maximize efficiency.

## 4 Conclusions

The extraction of cellulose from biomass using the organosolv method was efficient, as was the production of cellulose acetate by homogeneous acetylation. This was confirmed through FTIR, XRD and ^13^C NMR analyses. The produced cellulose acetate exhibited an acetylation degree of 2.64 ± 0.06 and a molar mass of 38,722 g mol^–1^. The successful synthesis of GO using the modified Hummers’ method and its functionalization with TiO_2_ through a solvothermal process were verified using FTIR, XRD, SEM and Raman spectroscopy analyses. The incorporation of GO and GO-TiO_2_ in the cellulose acetate membrane was confirmed by OM and SEM analyses, revealing that TiO_2_ functionalization improved the compatibility of GO with cellulose acetate. Filtration tests using the membranes highlighted important aspects about their permeability and selectivity against oil/water emulsions. The pure cellulose acetate membrane demonstrated acceptable performance in terms of oil rejection but showed fouling problems. The addition of GO significantly increased the permeate flux, especially at 0.5%, but the selectivity was compromised due to the detachment of GO particles, creating areas of low selective control. Compared with membranes containing GO, membranes with GO-TiO_2_, although exhibiting lower flux, did not present fouling issues and offered superior rejection.

Comparing these results with the initial objectives of the study, it is evident that the developed membranes have potential for application in oil/water emulsion separation processes. They achieved the goal of creating a polymeric nanocomposite with good flux characteristics and selectivity. However, challenges such as the compatibility between the embedded materials and the polymer matrix remain and must be addressed to optimize membrane performance.

## Supporting Information Available

(PDF) The Supporting Information is available free of charge at https://pubs.acs.org/doi/10.1021/acsomega.4c05980.

Additional results are presented in the following figures: Figure S1—(a) Solution ^1^H NMR spectrum of cellulose acetate. (b) Solution ^13^C NMR and solid-state ^13^C DP/MAS NMR spectra of cellulose acetate. Figure S2—(a)TGA and (b) DTG curves of GO and GO-TiO_2._ Figure S3—Pure water flux (F_w_) through the cellulose acetate membrane and the membranes incorporated with GO and GO-TiO_2_ (PDF)
