# Supplementary material for: Biomass-Derived Cellulose Acetate Membranes Modified with TiO2/Graphene Oxide for Oil-In-Water Emulsion Treatment
Source: ACS Omega. 2024 Sep 16;9(39):40882–96. doi: 10.1021/acsomega.4c05980 (PMC11447850; doi:10.1021/acsomega.4c05980)
Supplement: Supplementary file 1 — ao4c05980_si_001.pdf [file ao4c05980_si_001.pdf]

Supporting Information

Biomass-derived cellulose acetate membranes modified with TiO_2/graphene oxide for oil-in-water emulsion treatment

Djanyna V. C. Schmidt[a,*] (djanyna.schmidt@edu.ufes.br ; djanynaschmidt@gmail.com), Tainara L. G. Costa[a] (guerratainara@gmail.com), Daniel F. Cipriano[a] (daniel.cipriano@ufes.br), Carla S. Meireles[b] (carla.meireles@ufes.br), Cleocir J. Dalmaschio[c] (cleocir.dalmaschio@ufes.br), Jair C. C. Freitas[a] (jair.freitas@ufes.br)

[a] Laboratory of Carbon and Ceramic Materials, Department of Physics, Federal University of Espírito Santo, 29075-910, Vitória, ES, Brazil

[b] Laboratory of Advanced Materials, Department of Natural Sciences, Federal University of Espírito Santo, 29932-540, São Mateus, ES, Brazil

[c] Laboratory of Polymers, Department of Chemistry, Federal University of Espirito Santo, 29075-910, Vitória, ES, Brazil

*Corresponding author

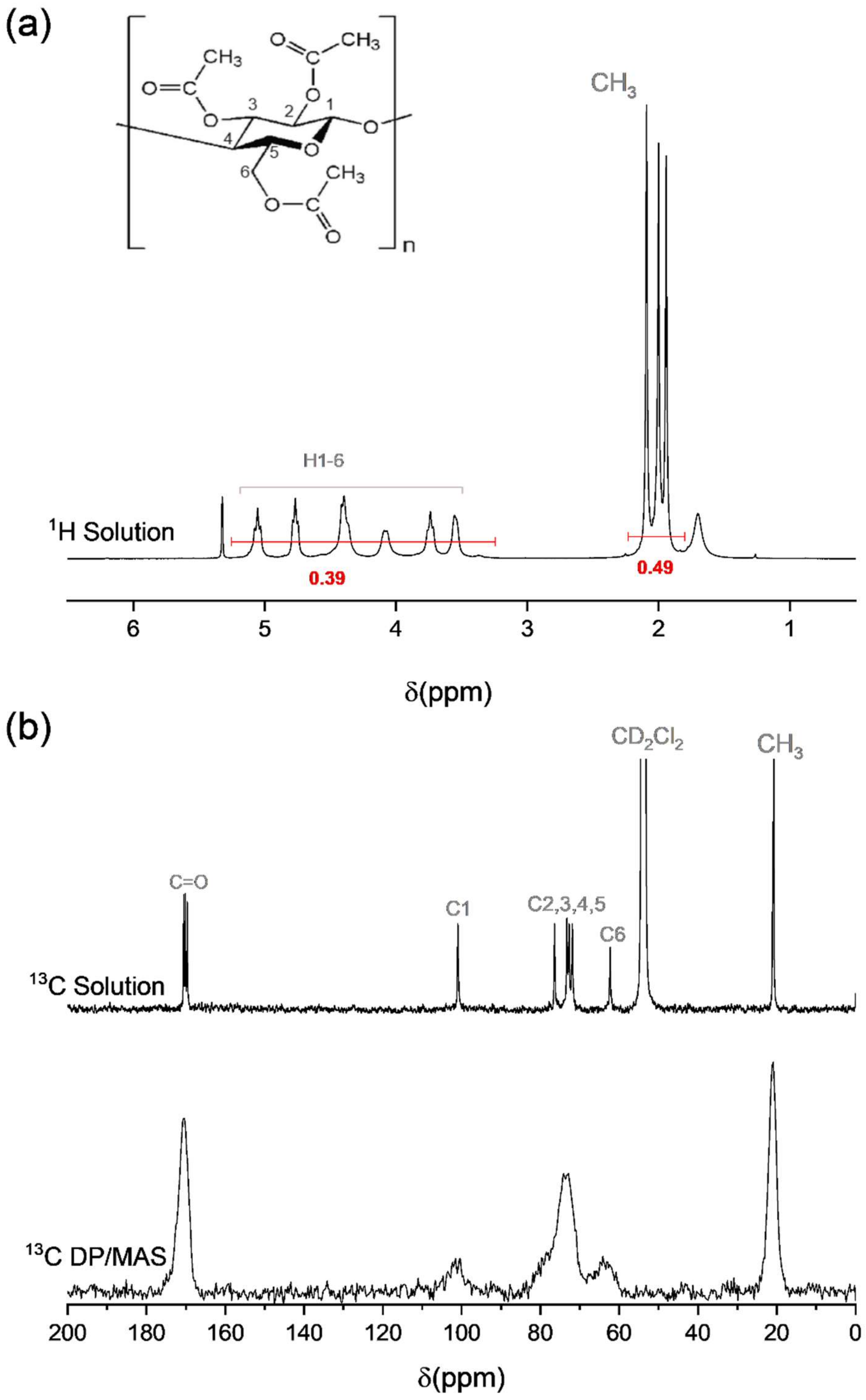

Fig. S1 – (a) Solution ^1H NMR spectrum of cellulose acetate. The values of the integrated areas corresponding to the indicated spectral regions are informed. (b) Solution ^{13}C NMR and solid-state ^{13}C DP/MAS NMR spectra of cellulose acetate.

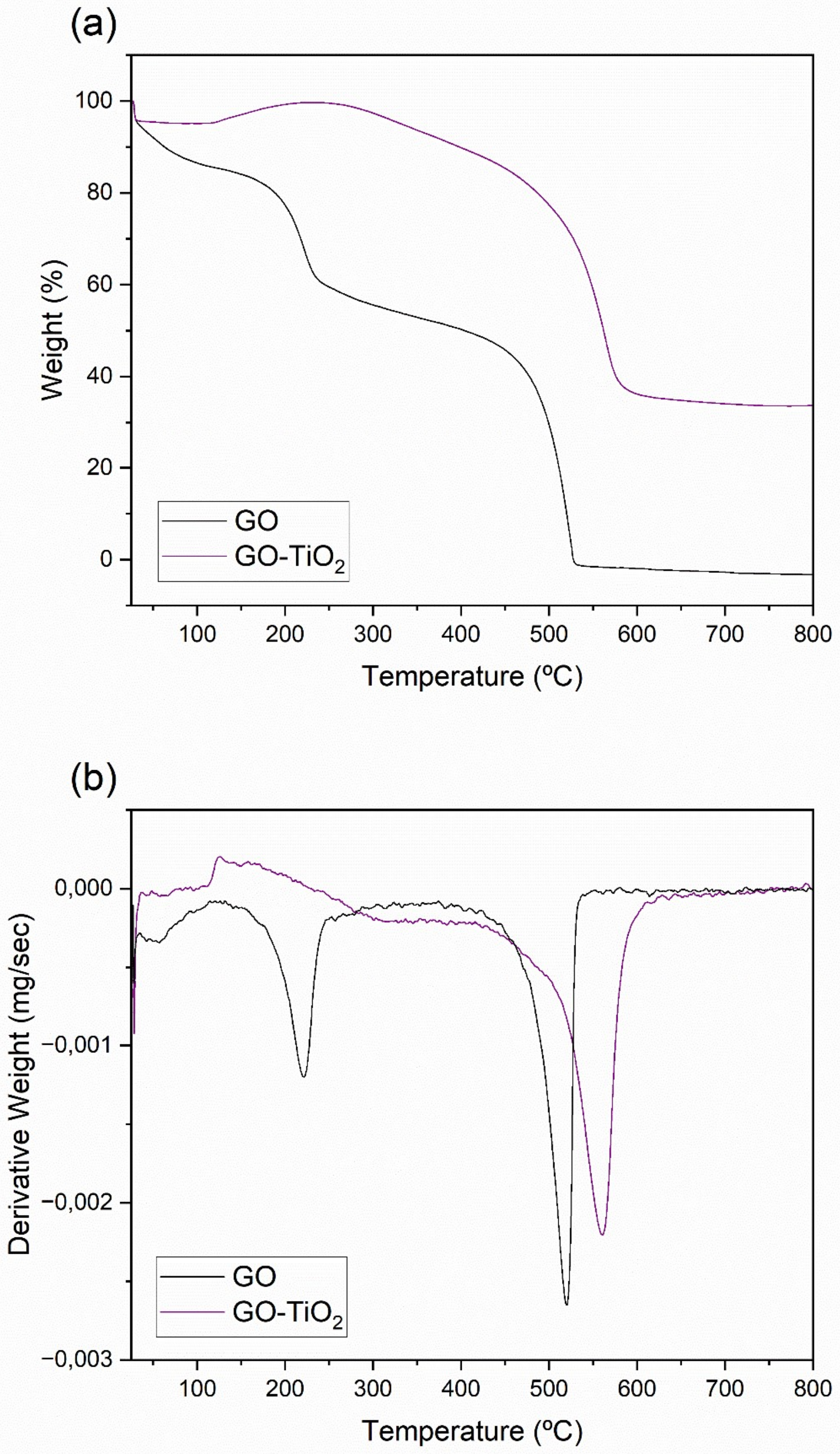

Fig. S2 – (a) TGA and (b) DTG curves of GO and GO-TiO_2.

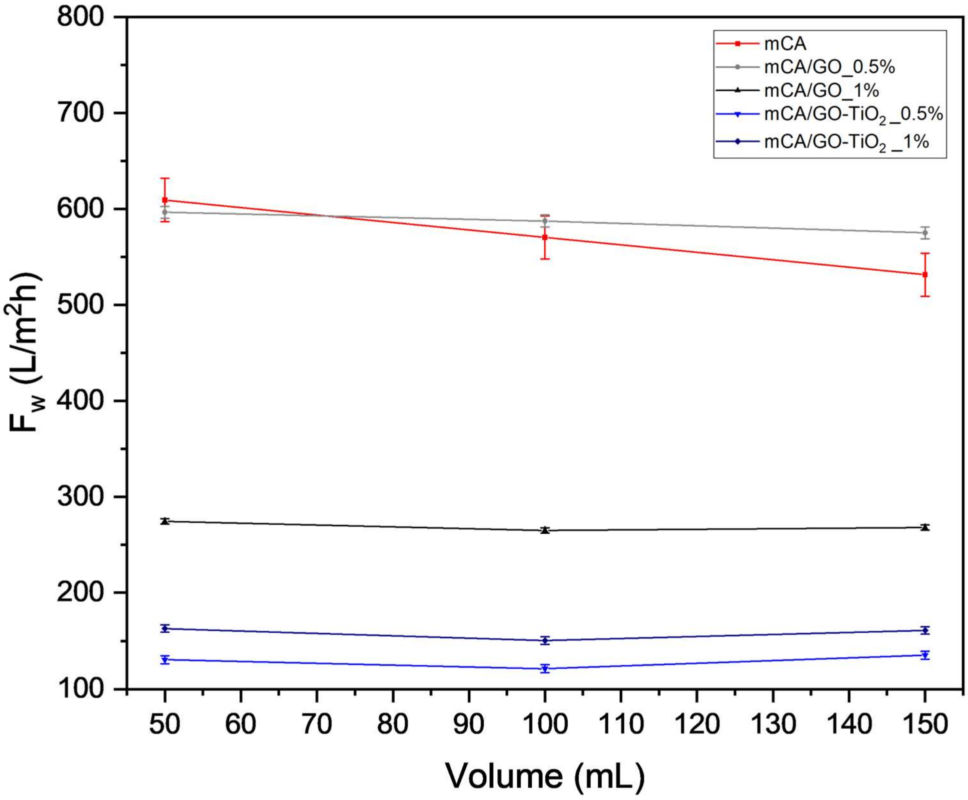

Fig. S3 – Pure water flux (F_w) through the cellulose acetate membrane and the membranes incorporated with GO and GO-TiO_2.
